# Diagnostic Performance of Sarcopenia Screening Tests in Chronic Lung Disease Patients

**DOI:** 10.5152/eurasianjmed.2025.25806

**Published:** 2025-04-17

**Authors:** Aslı Görek Dilektaşlı, Demet Kerimoğlu, Ayten Odabaş, Abdurrahman Doğan, Arzu Özpehlivan, Nilüfer Aylin Acet Öztürk, Özge Aydın Güçlü, Ezgi Demirdöğen, Funda Coşkun, Ahmet Ursavaş, Esra Uzaslan, Mehmet Karadağ

**Affiliations:** Department of Pulmonary Medicine, Bursa Uludağ University Medical School, Bursa, Türkiye

**Keywords:** Sarcopenia, chronic obstructive pulmonary disease, interstitial lung disease, Ishii test, SARC-F, SARC-CalF

## Abstract

**Objective:**

Sarcopenia, the gradual decline in skeletal muscle mass (SMM), strength, and functionality, has negative health consequences such as premature death and disability. It is prevalent in chronic lung disease (CLD). Timely recognition of sarcopenia is required for focused therapy. This study sought to analyze the rate of sarcopenia in patients with CLD and to assess the diagnostic accuracy of the sarcopenia screening tests: the SARC-F, SARC-CalF, and Ishii tests.

**Materials and Methods:**

This study comprised individuals diagnosed with CLD and referred for pulmonary rehabilitation. Sarcopenia was evaluated based on the European Working Group on Sarcopenia in Older People criteria (EWGSOP and EWGSOP2), utilizing handgrip strength, SMM index, and gait speed. The diagnostic accuracy of screening tests (SARC-F, SARC-CalF, and Ishii) was assessed by sensitivity, specificity, and the area under the curve (AUC) in the Receiver Ooperating Ccharacteristics.

**Results:**

A total of 227 patients, with a mean age of 59.00 ± 13.98 years, of whom 50.7% had chronic obstructive pulmonary disease (COPD), were included. The rate of probable sarcopenia was 41.2%, confirmed sarcopenia 2.5%, and severe sarcopenia 0.5%. The Ishii test exhibited the highest sensitivity (71.59%) and specificity (90.48%) for probable sarcopenia (AUC: 0.810); it also showed 100% sensitivity and substantial specificity (78.57%, AUC: 0.893) for confirmed sarcopenia.

**Conclusion:**

Sarcopenia is highly prevalent in CLD patients, underscoring the need for routine screening. Among the screening tools, the Ishii test exhibited the highest diagnostic accuracy, making it a valuable tool for early detection. Routine assessment and targeted interventions for sarcopenia could improve functional outcomes in CLD patients.

**Main Points**

- Despite its significant clinical implications, sarcopenia remains underdiagnosed in CLD due to a lack of routine screening.
- Our study found that 41.2% of CLD patients had possible sarcopenia, while 2.5% had confirmed sarcopenia, emphasizing the need for systematic assessment in this population. For screening, the Ishii test demonstrated the highest diagnostic accuracy, with superior sensitivity (71.59%) and specificity (90.48%) for probable sarcopenia. It also exhibited 100% sensitivity for confirmed sarcopenia, making it the most effective screening tool among those evaluated.
- Our findings emphasize that sarcopenia should be routinely assessed in CLD patients, particularly those undergoing pulmonary rehabilitation, where interventions can be tailored to address muscle loss and functional impairment.

## Introduction

Sarcopenia is a gradual loss of skeletal muscle strength, mass, and function, leading to a higher risk of impairment in mobility, falls, fractures, and mortality.^1^ Initially defined by the European Working Group on Sarcopenia in Older People (EWGSOP) based on muscle mass alone, the definition has since evolved to prioritize muscle strength, as it is a more robust predictor of negative outcomes.^1^ Muscle quality, which reflects structural and compositional changes, is also compromised in sarcopenia; however, routine assessment remains challenging due to technological limitations. Additionally, low physical performance is a key indicator of sarcopenia severity, as it is closely linked to functional decline and poor clinical outcomes.^1^

Although sarcopenia has traditionally been linked to aging, research now indicates that it can develop earlier in life, influenced by a range of factors beyond natural aging processes.^2^ Although body mass index (BMI) is a popular tool to evaluate nutritional health, it fails to adequately represent body composition.^3^ Recent studies have deepened our understanding by showing the presence of a subgroup of chronic obstructive pulmonary disease (COPD) patients who have a normal BMI, but whose muscle mass is disproportionately low.^3^ Traditionally, low muscle mass in COPD has been identified using the fat-free mass index.^4^ By understanding the importance of skeletal muscle mass (SMM) and quality, these criteria were initially designed to detect sarcopenia, a condition characterized by progressive muscle wasting.^1^,^5^

Chronic obstructive pulmonary disease is a predominant global health issue and the third foremost cause of mortality worldwide.^6^ Beyond pulmonary impairment, COPD is associated with systemic complications, including skeletal muscle dysfunction, osteoporosis, and cardiovascular disease.^7^ Nutritional status plays a pivotal role in these extrapulmonary effects, with sarcopenia and malnutrition in COPD patients linked to worse survival rates and a higher risk of exacerbations.^8^

Sarcopenia is now recognized as a valuable biomarker and prognostic factor in chronic lung disease (CLD) management, making it a relevant consideration in clinical practice. Its prevalence in asthma suggests a potential impact on lung function, airway obstruction, and comorbidities.^9^ In chronic fibrosing interstitial lung disease (ILD), both the underlying lung pathology and physical deconditioning contribute to muscle loss and poor outcomes. Studies indicate that patients with advanced CLD often exhibit reduced muscle mass and strength, emphasizing the complex interplay between sarcopenia, malnutrition, and metabolic dysfunction. Several cohort studies and case series have explored the prevalence and clinical implications of sarcopenia in ILD, underscoring its significance in disease progression and patient outcomes.

A variety of screening tools and diagnostic tests are available to assess sarcopenia in both clinical and research settings, with selection depending on patient characteristics, healthcare resources, and the purpose of evaluation.^1^,^10^ In clinical practice, early detection often begins with case-finding based on symptoms such as weakness, frequent falls, slow walking speed, difficulty standing from a seated position, or muscle wasting.^1^ Screening tools provide a non-invasive and cost-effective approach for identifying individuals at risk. The commonly used screening tools are SARC-F and extended version of it, the SARC-CalF.^11^^,12^ Ishii test estimates the probability of sarcopenia depending on age, grip strength, and calf circumference.^13^

This study aims to evaluate the prevalence of sarcopenia in the CLD group referred for pulmonary rehabilitation at our center. The performance of commonly used sarcopenia screening tools, including the SARC-F, SARX-CalF, and Ishii test, was assessed in a population with advanced CLD.

## Materials and Methods

### Study Participants

This study involved patients referred for pulmonary rehabilitation and diagnosed with a chronic pulmonary disease, from July 2017 to December 2023. Participants were eligible if they were 18 years or older and had a confirmed diagnosis of CLD such as COPD, asthma, bronchiectasis, ILD, or a history of lung cancer with resection surgery. Patients with neuromuscular disorders, end-stage organ failure, severe neurological deficits, lower limb amputations, or other conditions preventing safe ambulation were eliminated from the study. The study received approval from the Ethics committee of Bursa Uludağ University (Approval No: 2011-KAEK-26/980, Date: 12.12.2023). Study participants provided informed consent. Baseline demographic and clinical parameters, including age, sex, BMI, smoking history (pack-years), and lung disease diagnosis, were documented retrospectively from our pulmonary rehabilitation center records. Spirometry was conducted in accordance with the American Thoracic Society/European Respiratory Society (ATS/ERS) recommendations with a standardized spirometer.^14^ Forced expiratory volume in 1 second (FEV₁, Liter), forced vital capacity (FVC, Liter), and FEV₁/FVC ratio (%) were measured.

### Anthropometric Measurements

Anthropometric data, including height, weight, and calf circumference, were obtained using standardized techniques. The calf circumference was measured at its broadest position while the patient was seated, with the knee relaxed at a 90-degree angle of flexion and the foot flat on the floor, using a non-elastic measuring strip. All measurements were documented to the nearest 0.1 cm according to approved protocols.^15^ The BMI was calculated using the formula: weight (kg) divided by height squared (m^2^).

### Assessment of Muscle Mass and Muscle Strength

Skeletal muscle mass was evaluated using a whole-body bioelectrical impedance analysis (BIA) instrument (TANITA SC330, TANITA Health Equipment Hk Ltd, Hong Kong), with values adjusted for body height to assess muscle mass adequacy. The determination of SMM via bioelectrical impedance study was conducted using the equation formulated from a Caucasian population, as delineated by Janssen and associates.^16^ Skeletal muscle mass was normalized for body size and the skeletal mass index (SMI) was computed as SM/height^2^ (kg/m^2^). We used the following cut-off values to define low SMI: ≤5.75 kg/m^2^ for women and ≤8.50 kg/m^2^ for men, related to severe sarcopenia.^10^,^17^

Muscle strength was evaluated using isometric handgrip strength (HGS) assessment.^18^ Handgrip strength was evaluated by a hand dynamometer (Takei 5401 Digital Handgrip Dynamometer, Takei, Niigata-City, Japan) as a measure of muscle strength. Patients were directed to sit comfortably with their elbow relaxed at 90 degrees’ flexion, forearm in a neutral stance, and the wrist in mild extension (about 15 degrees). The assessment was conducted on the dominant hand, with patients instructed to exert maximum effort while squeezing the dynamometer for three trials, with one minute of rest between each session. The average value (kilograms) derived from the 3 trials was documented and utilized for analysis. This method is validated and is recommended for assessing physical performance and predicting adverse health outcomes in various clinical settings.^19^,^20^ Low muscle strength was described as less than 16 kg in women and less than 27 kg in men.^1^,^2^,^10^,^18^

### Physical Performance and Measurement of Gait Speed

The 4-m gait speed (4MGS) test was conducted to evaluate physical performance. The attending physician measured gait speed with a stopwatch as patients walked at their usual speed along a 4-m test course, outlined with start and end points in a flat corridor.^21^ Participants were directed to walk at a comfortable, normal pace, and the time taken to complete the course was recorded in seconds. The key outcome measure was gait speed, which was calculated by dividing the distance (4 m) by the elapsed time. A walking speed of less than 0.8 m/s indicated low physical performance.^1^,^10^,^18^,^22^

### Sarcopenia Definition and Classification

Sarcopenia was classified based on the EWGSOP criterion.^1^,^10^ *Probable sarcopenia* is characterized by the presence of low muscle strength. *Confirmed sarcopenia* is identified when low muscle strength is accompanied by low muscle mass. *Severe sarcopenia* is defined when both of these criteria are met along with low physical performance.^1^

### Screening Tests for Sarcopenia

The SARC-F questionnaire comprises 5 measures evaluating strength, walking ability, chair-raise ability, stair climbing, and fall history.^23^ Each item is rated on a scale from 0 to 2, with higher ratings signifying increased functional impairment. A score of ≥4 indicates a positive screening result for sarcopenia.^24^ We used the validated Turkish version of the SARC-F questionnaire.^24^ The SARC-CalF questionnaire extends SARC-F by adding the circumference of the calf as a sixth component.^25^ The scoring system for the initial five items is consistent with SARC-F, while the calf circumference item is assigned 0 points if the circumference exceeds the specified threshold (>33 cm) and 10 points if it is below 33 cm.^26^ A total SARC-CalF score of ≥11 signifies a positive screening for sarcopenia.

The Ishii screening test is an additional formal instrument for identifying cases of sarcopenia.^1^ The Ishii test assesses the likelihood of sarcopenia by incorporating age, grip strength, and calf circumference, as the scoring methodology is described by Ishii et al.^13^ A score of >105 in men and >120 in women is indicative of sarcopenia.^13^

### Statistical Analysis

Data were analyzed with IBM SPSS (IBM SPSS Corp.; Armonk, NY, USA) Statistics version 21.0. Descriptive statistics were applied to summarize patient demographics, nutritional status, frailty, activities of daily living (ADL) scores, and gait speeds. Categorical variables were defined using proportions. Continuous variables were presented as means and SD for data with a normal distribution, while medians and interquartile ranges (IQR 25-75) were utilized for non-normally distributed data. The diagnostic performance of SARC-F, SARC-CalF, and the Ishii screening test for identifying probable and confirmed sarcopenia was assessed based on four key performance metrics: sensitivity, specificity, positive predictive value (PPV), and negative predictive value (NPV). Receiver operating characteristic (ROC) analysis was performed to assess diagnostic accuracy, and the area under the curve (AUC) was computed. Area under the curve values were classified as follows: over 0.9 signifies good accuracy, 0.7-0.9 denotes moderate accuracy, 0.5-0.7 indicates low accuracy, and 0.5 implies a result comparable to random chance.

## Results

Included in the study were 227 participants, whose mean age was 59.00 ± 13.98 years. The predominant demographic of participants consisted of males (163 men, 64 women). A mean BMI of 26.37 ± 5.87 kg/m^2^ was recorded. Among the study participants, 115 (50.67%) were diagnosed with COPD, 7 (3.08%) with asthma, 22 (9.69%) with bronchiectasis, 69 (30.39%) with ILD, and 14 (6.17%) had undergone lung cancer resection surgery. About 64.3% of the study population were ex-smokers, while 27.2% and 8.5% were never and current smokers, respectively. The results of pulmonary function tests demonstrated a mean FEV₁ of 1.55 ± 0.68 L, an FVC of 2.76 ± 0.93 L, and an FEV₁/FVC ratio of 58.24 ± 21.27%, indicating varying degrees of airflow limitation across participants.Study population characteristics are summarized in Table 1.

### Muscle Strength and Physical Performance

Skeletal mass index was 9.68 ± 1.63 kg/m^2^, and HGS was 25.39 ± 8.44 kg on average. Calf circumference, an indicator of muscle mass, was 35.79 ± 4.37 cm. Among the 72 patients with available SMI data, 48.6% had normal SMI, 41.7% had moderate impairment, and 9.7% had high-degree impairment. Among the 216 patients with available HGS data, 58.8% had normal HGS, while 41.2% had low HGS.

Study participants had a mean 4-meter gait speed of 1.11 ± 0.80 m/s. Among the 165 patients with available gait speed data, 87.3% had normal gait speed, while 12.7% had slow gait speed, with a speed of ≤0.8 m/s.

### Sarcopenia Screening Tests

The median SARC-F total score was 3.00 (1.00-4.00), while the SARC-CalF total score was also 3.00 [1.00 – 4.00], suggesting that a significant proportion of participants exhibited sarcopenia-related symptoms. The Ishii test total score was 93.68 ± 35.93.

Among the 214 patients with an available Ishii score, 35% (n = 75) had scores indicative of sarcopenia. Similarly, among the 84 patients with a SARC-F score, 38.1% (n=32) met the criteria for sarcopenia, while 25% (n = 21) of those with a SARC-CalF score had results suggestive of sarcopenia (*data not shown*).

### Sarcopenia Prevalence

Among the 216 participants with available HGS measurements, 89 (41.2%) were classified as having *probable sarcopenia* due to low HGS. Among the 160 participants with both HGS and skeletal muscle index data, 4 (2.5%) met the criteria for *confirmed sarcopenia*, exhibiting both low HGS and reduced SMI below the defined cutoff values. Additionally, 1 case (0.5%) was classified as *severe sarcopenia*, defined by diminished muscle strength, reduced muscle mass, and gait speed.

### Probable Sarcopenia

The diagnostic performance of screening tests in detecting probable sarcopenia is summarized in Table 2. The SARC-F questionnaire demonstrated moderate specificity (74.47%) but relatively lower sensitivity (55.56%) in identifying probable sarcopenia based on HGS. PPV is 62.5%, meaning that 62.5% of individuals testing positive truly have probable sarcopenia. The NPV of 68.63% suggests that a negative result provides moderate confidence in ruling out sarcopenia.

These results indicate that SARC-CalF has lower sensitivity but higher specificity, meaning it is more effective at ruling out sarcopenia than detecting it. The PPV of 61.9% suggests that 61.9% of individuals testing positive truly have probable sarcopenia, while the NPV of 62.9% indicates that a negative result provides moderate confidence in ruling out the condition.

In contrast, the Ishii test has high sensitivity and specificity, making it effective at both detecting sarcopenia and correctly identifying non-sarcopenic individuals. The PPV of 84% suggests that a positive Ishii test score is strongly predictive of sarcopenia, while the NPV of 82.01% indicates that a negative result provides good confidence in ruling out sarcopenia.

**Figure 1.**
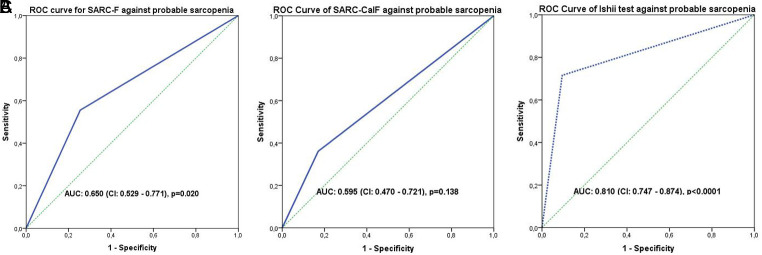
The ROC curves of SARC-F (A), SARC-CalF (B), and Ishii test (C) against EWGSOP probable sarcopenia criteria.

The ROC curves show that the Ishii test outperforms both SARC-F and SARC-CalF in detecting probable sarcopenia, with an AUC of 0.810 (*P* < .0001) compared to SARC-F (AUC: 0.650, *P* = .020) and SARC-CalF (AUC: 0.595, *P* = .138), indicating superior diagnostic accuracy, Figure 1A-C.

### Confirmed Sarcopenia

When assessing confirmed sarcopenia, SARC-F has moderate sensitivity and specificity in detecting confirmed sarcopenia. However, the PPV of 10.71% suggests that a positive test result has a low probability of correctly identifying confirmed sarcopenia, whereas the NPV of 98.08% indicates that a negative result is highly reliable in ruling out the condition.

SARC-CalF has moderate sensitivity and specificity in detecting confirmed sarcopenia, 75.00% and 67.11%, respectively. The PPV of 15.79% suggests that a positive result has limited predictive value, while the NPV of 98.36% indicates that a negative result is highly reliable in ruling out sarcopenia.

**Figure 2.**
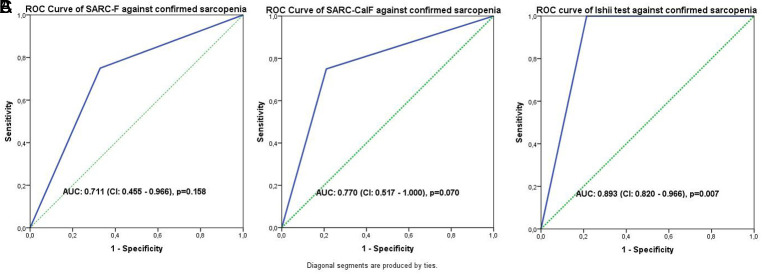
The ROC curves of SARC-F (A), SARC-CalF (B), and Ishii test (C) against EWGSOP confirmed sarcopenia criteria.

Ishii test has perfect sensitivity, meaning it correctly identifies all cases of confirmed sarcopenia. The specificity of 78.57% suggests a strong ability to rule out non-sarcopenic cases. However, the PPV of 10.81% suggests that a positive result has limited predictive value, while the NPV of 100.00% confirms that a negative Ishii test result completely rules out confirmed sarcopenia. Table 3 summarizes the diagnostic performance of screening tests in detecting confirmed sarcopenia in patients with chronic pulmonary disease. The ROC curves demonstrate that the Ishii test achieves the highest diagnostic accuracy for detecting confirmed sarcopenia, with an AUC of 0.893 (*P* = .007), outperforming SARC-F (AUC: 0.711, *P* = .158) and SARC-CalF (AUC: 0.770, *P* = .070), Figure 2A-C.

## Discussion:

This study assessed the diagnostic performance of screening instruments (SARC-F, SARC-CalF, and the Ishii test) and the prevalence of sarcopenia in patients with CLD who were referred for pulmonary rehabilitation. Our findings demonstrated that sarcopenia is common in this population, with 41.2% exhibiting probable sarcopenia, 2.5% confirmed sarcopenia, and 0.5% severe sarcopenia. Moreover, we noted that the Ishii test had superior sensitivity and specificity compared to other screening instruments, positioning it as a potentially valuable tool for sarcopenia screening in patients with CLD.

The high rate of probable sarcopenia we observed in this study aligns with previous research emphasizing skeletal muscle impairment in CLD. A very recent longitudinal study identified a significant correlation between probable sarcopenia and sarcopenia and an increased risk of CLD.^27^ Individuals diagnosed with either probable sarcopenia or sarcopenia were found to have a markedly higher likelihood of developing CLD.^27^ Wang et al observed an inverse correlation between muscle mass and the risk of developing CLD, suggesting that sarcopenia may be a modifiable factor in CLD management. Previous research confirmed a positive correlation between muscle mass and pulmonary function in elderly individuals, highlighting the potential role of muscle preservation in maintaining respiratory health. In our study population, the rates of probable and confirmed sarcopenia were 41.2% and 2.5%, underscoring its clinical relevance. This finding necessitates a dual perspective: first, recognizing the possible contribution of sarcopenia in chronic pulmonary disease development, and second, considering sarcopenia as a treatable trait in CLD management. Addressing muscle loss in CLD patients could offer a valuable therapeutic approach to improving functional outcomes and disease progression.

In the general aging population, sarcopenia, as defined by these criteria, has a prevalence of approximately 15%.^28^ Current findings in the literature show that sarcopenia prevalence is increased in CLD populations. The catabolic effects of chronic inflammation, reduced physical activity, malnutrition, and systemic steroid use are known contributors to muscle wasting in pulmonary diseases.^5^ Studies indicate a significant prevalence of sarcopenia in COPD patients, ranging from 15.5% to 34%, with the frequency increasing as disease severity progresses.^29^^-^ ^31^ Research demonstrates that sarcopenia is common in patients with ILD, with prevalence rates between 22.9% and 39%, dependent upon the cohort.^32^,^33^ A meta-analysis estimated that 26% of IPF patients fulfill the EWGSOP2 sarcopenia definition, which is similar to COPD but much greater than that of the general population.^34^ Research in ILD has highlighted low muscle mass and reduced strength, which contribute to worse functional outcomes and disease progression.^35^ The sarcopenia prevalence in asthma patients varies from 12% to 21%.^9^ The correlation between sarcopenia and asthma prevalence, lung function, and associated comorbidities is a less studied area of research. Hu and colleagues^9^ observed that sarcopenia was related with an increase in asthma-related symptoms. Additionally, sarcopenia, particularly severe sarcopenia, was related with higher risk of airway obstruction.^9^ Furthermore, sarcopenia showed a positive association with depression and COPD, highlighting its potential role in asthma-related comorbidities.^9^ Sarcopenia may play a role in asthma development by impacting lung function and comorbidities, making it a potentially modifiable factor in asthma management. Given its high prevalence and significant impact, routine screening for sarcopenia in individuals with asthma could be beneficial for early detection and intervention. Significant reduction in muscle mass and body composition parameters of bronchiectasis patients were evident.^36^,^37^

Our findings indicate that sarcopenia screening tests exhibit varying levels of diagnostic performance in detecting sarcopenia. The SARC-F questionnaire, widely used due to its simplicity, demonstrated moderate specificity (74.47%) but lower sensitivity (55.56%), limiting its effectiveness in identifying early sarcopenia cases. These results are in line with previous studies that reported SARC-F’s limited sensitivity in chronic disease populations.^23^ The SARC-CalF, which incorporates calf circumference, exhibited higher specificity (82.98%) but lower sensitivity (36.11%), making it more useful in confirming rather than detecting sarcopenia.^11^ Bahat and colleagues^11^ assessed the diagnostic performance of sarcopenia screening tests in a Turkish population consisting of a community-dwelling elderly population. They found the sensitivity and specificity as 25% and 81.4% for SARC-F; 25.0% and 98% for SARC-CalF (CC < 31 cm) against EWGSOP criteria. Our analysis revealed that the specificity of both SARC-F and SARC-CalF aligns with Bahat's findings. We observed that SARC-F exhibits greater sensitivity in patients with CLD, indicating that it may serve as a more appropriate screening instrument for this population compared to older persons living in the community.^11^

In this study, the Ishii test demonstrated superior diagnostic performance, with high sensitivity (71.59%) and specificity (90.48%) in detecting probable sarcopenia. For confirmed sarcopenia, it achieved perfect sensitivity (100%) and high specificity (78.57%), outperforming both SARC-F and SARC-CalF. Consistent with our findings, Erdogan et al^38^ documented sensitivity as 84%, 100%, and 100%, and specificity as 86.1%, 83.9%, and 84.6% for probable, confirmed, and severe sarcopenia, respectively, in community-dwelling older adults. These findings indicate that the Ishii test may serve as a more effective screening instrument for identifying sarcopenia in CLD populations, an observation supported by other studies highlighting its strong diagnostic accuracy.^38^^,^^39^

This study has several limitations. First, muscle mass was evaluated by bioelectrical impedance analysis, a validated and feasible method, is less precise than dual-energy X-ray absorptiometry for assessing body composition, fat-free mass, and appendicular skeletal muscle mass (ASM).^40^ Current recommendations suggest ASM and appendicular skeletal muscle mass index (ASMI) as the optimal metrics for diagnosing confirmed sarcopenia, but our available equipment did not allow for these measurements^1^,^10^ and accordingly did not calculate the ASMI.^41^ Instead, we used SMI cutoffs previously defined by Janssen et al^17^ to estimate muscle loss risk. The current recommendations suggest the measurement of ASM and ASMI as the select of choice parameters for confirming sarcopenia.^42^ Despite its portability, accessibility, and prevalent use in clinical environments, BIA has limits, especially in persons with excessive body mass, whose reference equations may lack comprehensive validation. Furthermore, BIA is unable to evaluate particular muscle areas or muscle quality, which are widely acknowledged as critical components in the diagnosis of sarcopenia.^43^ Second, our sample size was moderate, potentially constraining the generalizability of our findings to larger groups with chronic pulmonary disease. Moreover, the number of patients in specific subgroups, such as COPD, asthma, and ILD, was relatively small. Additionally, the incidence of confirmed and severe sarcopenia was lower than expected, despite our study population consisting of individuals with advanced CLD referred for pulmonary rehabilitation. One potential reason for this discrepancy is the limited availability of SMI data, which was only accessible for approximately one-third of the participants. Future studies should incorporate bigger cohorts and employ improved imaging techniques for measuring ASMI to achieve a more thorough understanding of sarcopenia prevalence and severity in this population, hence improving diagnosis accuracy. Lastly, the retrospective methodology prevents causal inferences regarding the relationship between sarcopenia and pulmonary function and functional capacity decline. Future prospective and longitudinal studies are essential to investigate the role of sarcopenia in pulmonary disease progression, its impact on rehabilitation outcomes, and the potential benefits of targeted interventions in CLD management.

The high prevalence of sarcopenia in chronic pulmonary disease patients underscores the need for routine screening and early intervention. Given that low muscle mass and strength are associated with impaired lung function, poor exercise capacity, and increased mortality,^1^^,^^5^^,^^26^ incorporating sarcopenia assessment into pulmonary rehabilitation programs may improve patient outcomes. The Ishii test, given its high diagnostic sensitivity and specificity, may serve as an efficient screening tool in clinical settings, helping to identify patients who require targeted nutritional and exercise interventions for management of sarcopenia.^44^

In conclusion, this study highlights the high prevalence of sarcopenia in patients with CLD referred for pulmonary rehabilitation. Among the screening tools evaluated, the Ishii test demonstrated the highest accuracy, making it a valuable tool for sarcopenia detection. Routine screening for sarcopenia in this population is crucial, as early identification and targeted interventions may help improve functional outcomes and reduce disease burden.

## Figures and Tables

**Table 1.** Characteristics of the Study Population

	N = 227
Age, years	59.00 ± 13.98
Sex (M/F)	163/64
BMI, kg/m^2^	26.37 ± 5.87
*Chronic lung disease, N (%)*	
COPD	115 (50.67)
Asthma	7 (3.08)
Bronchiectasis	22 (9.69)
Interstitial lung disease	69 (30.39)
Lung cancer, resection surgery	14 (6.17)
*Muscle strength*	
Handgrip strength,^a^ kg	25.39 ± 8.44
*Muscle quantity*	
Skeletal mass index,^b^ kg/m^2^	9.68 ± 1.64
*Physical performance*	
4-meter gait speed,^c^ m/s	1.11 ± 0.80
*Pulmonary function testing^d^*	
FEV_1_, L	1.55 ± 0.68
FVC, L	2.76 ± 0.93
FEV_1_/FVC, %	58.24 ± 21.27
Calf circumference,^e^ cm	35.79 ± 4.37
Smoking history/pack-years	30.00 (0.00-40.00)
SARC-F total score^f^	3.00 (1.00-4.00)
SARC-CalF total score^f^	3.00 (1.00-4.00)
Ishii test score^g^	93.68 ± 35.93

Data are mean ± SD or median (interquartile range 25-75), as appropriate. Chronic lung disease distribution is presented in numbers (percentages).

FEV, Forced expiratory volume; F, female; FVC, forced vital capacity; kg, kilograms; M, male; m, meters; s, seconds

^a^Denotes data available for 216 participants. ^b^Denotes data available for 72 participants. ^c^Denotes data available for 165 participants. ^d^Denotes data available for 226 participants.^ e^Denotes data available for 221 participants. ^f^Denotes data available for 84 participants. ^g^Denotes data available for 214 participants.

**Table 2.** Diagnostic Performance of SARC-F, SARC-CALF, and Ishii Test in Diagnosing Probable Sarcopenia in Patients with a Chronic Pulmonary Disease

	SARC-F	SARC-CalF	Ishii Test
Sensitivity, %	55.56	36.11	71.59
Specificity, %	74.47	82.98	90.48
PPV, %	62.50	61.90	84.00
NPV, %	68,63	62.90	82.01
AUC, 95% CI	0.650 (0.529-0.771)	0.595 (0.470-0.721)	0.810 (0.747-0.874)

AUC, area under curve; CI, confidence interval; NPV, negative predictive value; PPV, positive predictive value.

**Table 3.** Diagnostic Performance of SARC-F, SARC-CalF, and Ishii Test in Diagnosing Confirmed Sarcopenia in Patients with a Chronic Pulmonary Disease

	SARC-F	SARC-CalF	Ishii Test
Sensitivity, %	75.00	75.00	100.00
Specificity, %	67.11	78.95	78.57
PPV, %	10.71	15.79	10.81
NPV, %	98.08	98.36	100.00
AUC	0.711 (0.455-0.966)	0.770 (0.517-1.000)	0.893 (0.820-0.966)

AUC, area under curve; NPV, negative predictive value; PPV, positive predictive value.

## Data Availability

The data that support the findings of this study are available on request from the corresponding author.
